# Acquired MTAP Loss Following Entrectinib Resistance in ROS1‐Rearranged NSCLC With CD74 Exon 3–ROS1 Exon 34 Fusion

**DOI:** 10.1111/1759-7714.70176

**Published:** 2025-10-21

**Authors:** Mizuha Haraguchi Hashiguchi, Maika Tanino, Suzuyuki Yoneda, Masato Asaoka, Junko Kagyo, Makoto Katayama, Hideki Terai, Kohei Nakamura, Hiroshi Nishihara, Koichi Fukunaga

**Affiliations:** ^1^ Division of Pulmonary Medicine, Department of Medicine Keiyu Hospital Yokohama Japan; ^2^ Department of Neurosurgery Kawasaki Municipal Hospital Kawasaki Japan; ^3^ Division of Pulmonary Medicine, Department of Medicine Keio University, School of Medicine Tokyo Japan; ^4^ Keio Cancer Center Keio University School of Medicine Tokyo Japan; ^5^ Center for Cancer Genomics Keio University School of Medicine Tokyo Japan

## Abstract

This case highlights acquired MTAP loss during disease progression in ROS1‐rearranged NSCLC. Despite persistent CD74–ROS1 fusion and absence of known resistance mutations, the patient developed CNS progression after entrectinib, underscoring the value of longitudinal genomic profiling in guiding treatment decisions.

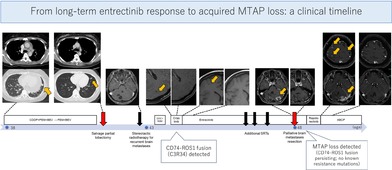

We previously reported a lung adenocarcinoma case in a 38‐year‐old never‐smoking female harboring a novel CD74 exon 3–ROS1 exon 34 (C3R34) fusion, demonstrating a marked response to entrectinib [1]. Here, we present the clinical course focusing on the emergence of acquired MTAP (methylthioadenosine phosphorylase) loss at age 48 despite persistent ROS1 fusion positivity.

At 43 years, after postoperative recurrence, comprehensive genomic profiling (FoundationOne) confirmed the CD74–ROS1 (C3R34) fusion [1]. Entrectinib treatment achieved durable disease control for approximately 4 years. During this period, a new cerebellar lesion appeared and was initially managed with stereotactic radiotherapy, but eventually progressed, causing cerebellar symptoms. Surgical resection at 48 years confirmed viable adenocarcinoma.

In‐house hybridization capture‐based CGP (PleSSision Rapid) [2] revealed ongoing CD74–ROS1 fusion. Importantly, no known secondary ROS1 resistance mutations (such as G2032R, D2033N, S1986F/Y, or L2026M), nor bypass pathway alterations (including EGFR, KRAS, BRAF, MET, and PIK3CA), were detected. However, newly acquired MTAP loss was identified. Given the clinical progression and available genomic data, therapy was switched to repotrectinib; however, the patient showed no clinical benefit and developed neurological deterioration with diplopia and abducens nerve palsy. MRI showed local recurrence, multifocal brain metastases, and leptomeningeal enhancement. Subsequent radiotherapy was given for symptom palliation, followed by ABCP chemotherapy (atezolizumab, bevacizumab, carboplatin, and paclitaxel). After four cycles, imaging demonstrated stabilization of irradiated lesions and regression of non‐irradiated metastases. A timeline of the clinical course is shown in Figure 1.

**FIGURE 1:**
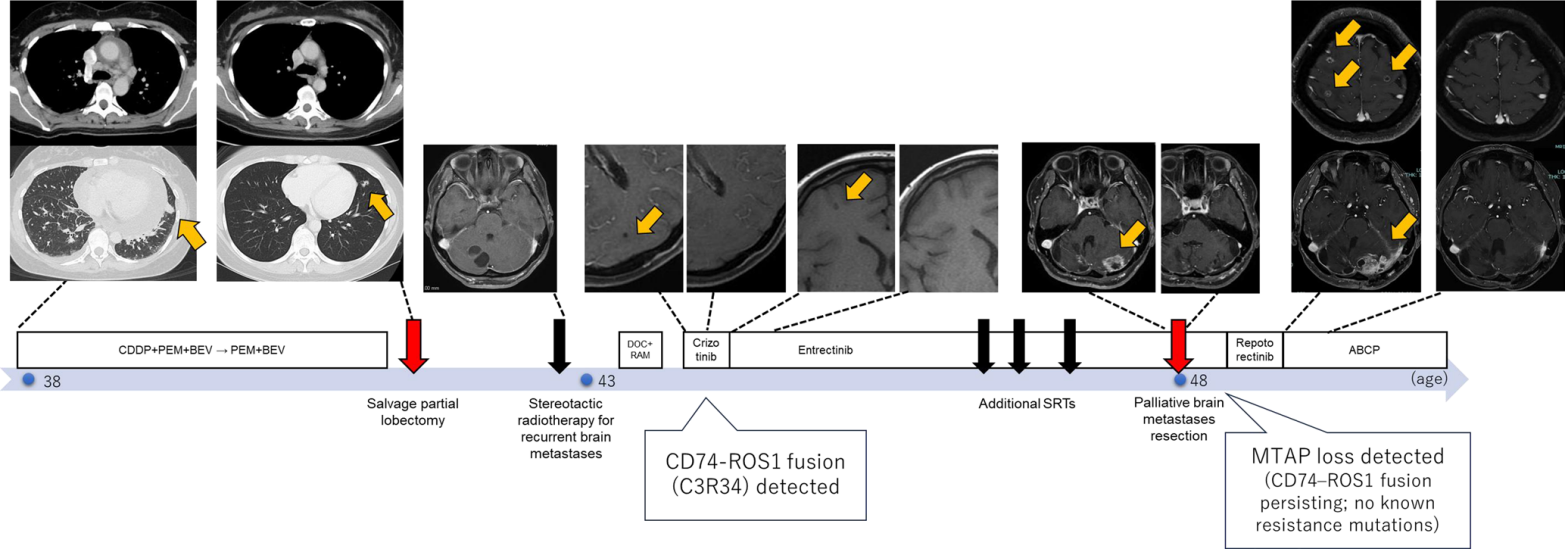
Timeline of the clinical course in a patient with CD74–ROS1‐rearranged NSCLC who acquired MTAP loss. At age 38, chest CT revealed the primary lung tumor in the left lower lobe (yellow arrow). At age 43, following recurrence, comprehensive genomic profiling (FoundationOne) confirmed a CD74–ROS1 fusion, and brain MRI showed new metastatic lesions (yellow arrows). At age 48, a cerebellar metastasis was surgically resected. In‐house genomic profiling (PleSSision Rapid) demonstrated persistent CD74–ROS1 fusion, no other known resistance mutations, and newly acquired MTAP loss. Subsequent MRI revealed local recurrence, multifocal brain metastases, and leptomeningeal enhancement (yellow arrows). Yellow arrows indicate tumor lesions. The lower panel illustrates the clinical timeline with patient age at key events. Abbreviations: ABCP, atezolizumab + bevacizumab + carboplatin + paclitaxel; SRT, stereotactic radiotherapy. From long‐term entrectinib response to acquired MTAP loss: A clinical timeline.

MTAP loss has been implicated in tumor aggressiveness and resistance mechanisms across cancers. This case raises the possibility that MTAP loss may be associated with disease progression under ROS1‐targeted therapy, although causality cannot be established. Importantly, MTAP deficiency may confer sensitivity to pemetrexed [3] and PRMT5 inhibitors [4], indicating possible therapeutic avenues in refractory ROS1‐positive NSCLC. This report is limited by its single‐case nature, which precludes generalizability. Moreover, while MTAP loss was detected at progression, this temporal association does not prove causality, and unrecognized resistance mechanisms may have contributed. Nevertheless, this case highlights that longitudinal molecular profiling of recurrent lesions, including CNS sites, may provide actionable insights for treatment selection in advanced NSCLC.

## Author Contributions

**Mizuha Haraguchi Hashiguchi:** conceptualization, writing – original draft, writing – review and editing. All other authors: writing – review and editing.

## Ethics Statement

The authors have nothing to report.

## Consent

Written informed consent was obtained from the patient for publication of all clinical and genomic information presented in this manuscript.

## Conflicts of Interest

The authors declare no conflicts of interest.

## Data Availability

The data that support the findings of this study are available from the corresponding author upon reasonable request. The data are not publicly available due to privacy or ethical restrictions.
